# Editorial: The burden of stress and depression – new insight into faster and efficient treatment

**DOI:** 10.3389/fnbeh.2022.998307

**Published:** 2022-08-08

**Authors:** Keren Nitzan, Matthew O. Parker, Alon Shamir, Gang Chen, Ravid Doron

**Affiliations:** ^1^Department of Education and Psychology, The Open University, Ra'anana, Israel; ^2^Institute for Biological and Biomedical Sciences, School of Pharmacy and Biomedical Science, University of Portsmouth, Portsmouth, United Kingdom; ^3^Faculty of Medicine, Technion–Israel Institute of Technology, Haifa, Israel; ^4^Mazor Mental Health Center, Akko, Israel; ^5^Interdisciplinary Institute for Personalized Medicine in Brain Disorders, Jinan University, Guangzhou, China

**Keywords:** depression, stress, herbal treatment, noradrenergic agents, ketamin, microbiome, acupuncture, anxiety

Depression and anxiety are prevalent disorders and a major public health concern worldwide. Although current treatments for depression and anxiety have some pronounced limitations—the development of new drugs for mood disorders has been at a standstill for the last decade. Moreover, there is currently no reliable biomarker for the early detection of mood disorders.

This Research Topic presents theoretical and experimental work of unique strategies to diagnose and treat anxiety and depression. It includes 13 articles: 9 original research, one review, two mini-reviews, and one opinion article. Most of the research presented here tries to tackle one of the more severe limitations of currently available treatments: prolonged onset time. Others explore non-conventional therapies for the treatment of depression and anxiety, such as Chinese traditional medicine, including herbs and acupuncture. The section also presents novel research regarding possible biomarkers and risk factors for depression, as well as an interesting view on the importance of DNA methylation in depression.

Current anti-depressants drugs enhance monoamine neurotransmitters, either by enzyme inhibition such as in Monoamine oxidase inhibitors (MAOIs) drugs, reuptake inhibition of both serotonin and adrenaline such as Tricyclic antidepressants (TCAs) and serotonin–noradrenaline reuptake inhibitors (SNRIs) or specifically blocking just the serotonin transporter such as Selective serotonin reuptake inhibitors (SSRIs).

The special topic review paper by Fitzgerald urges researchers to remember that although most of the drug-research industry is focused on drugs that boost synaptic monoamines, a significant body of studies suggests that noradrenergic transmission reducing drugs can be effective anti-depressants. Fitzgerald emphasizes the importance of the noradrenergic pathways as a target for new drugs, focusing on three major classes of noradrenergic transmission reducing drugs (alpha2 agonists, beta-blockers, alpha1 antagonists), and supports the hypothesis that they have antidepressant-like properties.

In their special topic paper, Bareli et al., give much-needed attention to depression and anxiety associated with substance use disorders (SUDs). They suggest a novel candidate for pharmacological treatment of patients with SUD and comorbid mood/anxiety disorders that may facilitate their rehabilitation. The authors tested, in both animals and patients, a novel combination of opipramol and baclofen (O/B), which is known to attenuate anxiety and depression, for the facilitation of recovery from SUDs. Their findings indicate a beneficial effect of O/B treatment.

## Fast onset drugs

One major limitation of current treatment for depression and anxiety is the slow onset of therapeutic action, with several weeks of therapy required before achieving a therapeutic response. Due to the slow-onset nature of current drugs, many patients experience long periods of depressive symptoms without being beneficially treated, and the delayed therapeutic effects lead to discontinuation of treatment (Srimongkon et al., 2018), and potentially putting patients at higher risk of suicide (Valenstein et al., 2009). Finding an accelerating agent for the current anti-depressant drugs will help to improve adherence, quality of life, productivity, and wellbeing of many patients.

Ketamine, a glutamate NMDA receptor channel blocker, can produce a fast and sustained anti-depressant response. The discovery of Ketamine is considered one of the most significant breakthroughs in the field of depression since the 1950s. The special topic paper by Colla et al. reviews mechanisms that may relate to Ketamine's anti-depressant effect. Colla et al. describe current theories of anti-depressant drug action, including monoaminergic signaling, disinhibition of glutamatergic neurotransmission, neurotrophic and neuroplastic effects, and discuss how these different mechanisms might relate to ketamine action. While Chen et al., in their research article, provide insight into the role of glutamate transporter 1 (GLT1) as the critical presynaptic molecule participating in the pathophysiological mechanism of depression and contributing to the antidepressant-like effect of Ketamine. They show that GLT1 expression levels in the Prefrontal Cortex significantly decrease in stressed mice and return to normal by ketamine treatment. Moreover, pretreatment with the GLT1 inhibitor DHK significantly alleviated the rapid antidepressant-like effect of ketamine infusion. Using specific inhibitors, Chen et al., confirm that both AMPA (α-amino-3-hydroxy-5-methyl-4-isoxazolepropionic acid) receptor and L-type voltage-dependent calcium channels (L-VDCC) are crucial factors in the immediate antidepressant-like effect of Ketamine.

While the central nervous system no doubt plays an integral role in developing anxiety and depression—it is not the only player. Recent evidence suggests a tight connection between the gut, the brain and psychiatric disorders (Mitrea et al., 2022). The special topic paper by Wilkowska et al. reviews the importance of the gut microbiome and gives special attention to the effect of Ketamine on the microbiome in animal models of depression. They present preliminary studies indicating that Ketamine restores bacteria-producing anti-inflammatory substances, reduces the number of bacteria associated with inflammatory processes in the gut, reduces the number of bacteria, previously reported as increased in depression, and increases the abundance of probiotic bacteria known to produce an antidepressant effect. Wilkowska et al. conclude by emphasizing the need for further studies on the effect of ketamine and its enantiomers on individual bacterial species.

While Ketamine is a promising candidate for treating depression and anxiety, it has some acute side effects, and more importantly, its chronic use is associated with potentially severe and possibly persistent toxic effects (Short et al., 2018). Thus, the search for a safe and side-effect-free treatment is still ongoing. Traditional Chinese medicines have a long history of treating mood disorders, some of which are still actively used today; thus, it has the potential to serve as a safe and effective alternative to conventional drugs and can be an alternative option for treatment (Burstein et al., 2021).

## Alternative and Chinese medicines

Kim R. Y. et al. demonstrated the anti-depressant effects of Fraxinus rhynchophylla Hance (*F. rhynchophylla* Hance, FX) in a reserpine-induced mouse model of depression. Ten-day treatment alleviated anxiety and depression like-behaviors, attenuated plasma corticosterone concentrations, decreased pro-inflammatory cytokines mRNA levels, and increased hippocampal phosphorylated cAMP response element-binding protein (pCREB) and brain-derived neurotrophic factor (BDNF). Kim R. Y. et al. findings serve as a preclinical basis to confirm the potential of FX as an anti-depressant drug; although, further studies are needed to establish its mechanisms of action.

Exploring a different Chinese herb, Zhang et al. show the beneficial effect of the Yueju pill in clinical trials. Yueju, a herbal medicine, has been shown to promote anti-depressant effects in many preclinical studies. In the special topic paper here, Zhang et al. present the beneficial effect of the Yueju pill, compared to either placebo or Escitalopram in two separate clinical trials. In a preliminary open-labeled trial on major depressive disorder (MDD) patients, they found symptom alleviation as early as 1 week post a conventional low dose of Yueju. In the confirmatory random controlled double-blinded clinical trial, they found both Escitalopram and Yueju pill resulted in early improvement of depression symptoms, and comparable antidepressant outcomes after 4 weeks of treatment.

Aside from pharmacological interventions, some patients with depression may prefer non-pharmacological options. A unique study, by Sakurai et al., explores for the first time difference in brain activation associated with relaxation effects of Autonomous sensory meridian response (ASMR) videos compared to classical music therapy. Their results show that classical music and the ASMR auditory stimulus produced a pleasant and relaxed state but that ASMR involves more complex brain functions than classical music, especially the activation of the medial prefrontal cortex.

Another alternative Chinese treatment that has emerged as a promising non-pharmacological treatment for reducing depressive symptoms is Acupuncture (Yang et al., 2022).

A meta-analysis by Jiang et al., compares acupuncture's effectiveness to other non-pharmacological treatments such as cognitive-behavioral treatment, mindfulness, behavioral activation program, brain electrical biofeedback therapy, tai chi, and bright light therapy (among others) on Sub-threshold depression (SD). Their results suggest that electroacupuncture and bright light therapy appear to be the better choices in the treatment of SD.

In agreement with this view, the special topic paper by Kawanokuchi et al., demonstrated acupuncture's effectiveness in preventing and treating the symptoms of social defeat stress (SDS)-induced depression in mice. Two weeks of acupuncture restored SDS-reduced brain-derived neurotrophic factor (BDNF), neurotrophin (NT)-3, and NT-4/5 expression. In contrast, acupuncture stimulation suppressed nerve growth factor (NGF) expression induced by SDS. The authors conclude that acupuncture treatment could effectively correct the imbalance in the expression of neurotrophic factors.

## Biomarkers and risk factors

BDNF is involved in the pathogenesis of mood disorders and has been associated with the action of anti-depressant and anxiolytic drugs (Colucci-D'Amato et al., 2020). Interestingly, in the previously mentioned clinical trial by Zhang et al., serum levels of BDNF, in combination with depression scores, yielded a new possible marker (i.e., “neuroplasticity index”) that may serve as a predictor for anti-depressant treatment outcome.

Indeed, there is a vital need for biomarkers that could predict response to anti-depressant drugs as more than one-third of patients (40%) do not respond to the anti-depressant treatment. The special topic paper by Vieira et al., tried to tackle this important issue. They found that alterations in white matter integrity, specifically in forceps minor and the superior longitudinal fasciculus, are associated with paroxetine treatment response. Although the authors acknowledge several limitations to their study, they offer a promising initial step forward in the path to discovering a reliable biomarker.

Many factors may be involved in the susceptibility to anxiety and depression, such as age, gender, early life stress and more (Mofatteh, 2020). The special topic paper by Kim S. et al. tries to determine whether genetic hypersensitivity to stress would alter behaviors in adulthood after limited mild stress during early adolescence and explore sex differences in response to stress in rats. While Lax, in his special topic paper, maintains that environmental factors, such as early-life stress, make individuals prone to major depression. He stipulates that these environmental factors modulate epigenetic signals to reprogram brain gene-expression patterns, ultimately affecting DNA methylation. Thus, DNA methylation may be used as a potential biomarker to predict MDD and its severity in vulnerable populations and treatment outcomes. Furthermore, Lax suggests that as drugs that modify DNA methylation are available and demonstrate significant effects across both preclinical and clinical studies, they have the potential to be used as adjuvants, increasing the efficacy of classic anti-depressant treatments.

In summary, current treatment for depression and anxiety suffers from major limitations—mainly major side effects and prolonged on-set time. Ongoing research is looking for new treatments, focusing on novel mechanisms both in the central nervous system and periphery—specifically the interesting connection between the gut and the brain. Furthermore, when looking for treatments one should also consider environmental factors and risk factors that can modify genetic processes, such as DNA methylation, and cause treatment resistance and increased susceptibility to anxiety and depression.

“Frontiers” Special topics offer a unique platform to explore innovative research on current matters and should be further continued, focusing on other specific issues such as the search for treatment without side effects and causes for treatment resistance. Overall, we believe that the contributions to the Special Topic “*The burden of stress and depression – new insight into faster and efficient treatment*” highlight the importance of exploring new venues for the treatment of depression and anxiety, and points to alternative herbal and non-pharmacological options; and suggest several possibilities for biomarkers and risk-factor to ultimately facilitate better care for patients.

## Author contributions

All authors listed have made a substantial, direct, and intellectual contribution to the work and approved it for publication.

## Conflict of interest

The authors declare that the research was conducted in the absence of any commercial or financial relationships that could be construed as a potential conflict of interest.

## Publisher's note

All claims expressed in this article are solely those of the authors and do not necessarily represent those of their affiliated organizations, or those of the publisher, the editors and the reviewers. Any product that may be evaluated in this article, or claim that may be made by its manufacturer, is not guaranteed or endorsed by the publisher.
